# Identification of Synergistic Interaction Between Cannabis-Derived Compounds for Cytotoxic Activity in Colorectal Cancer Cell Lines and Colon Polyps That Induces Apoptosis-Related Cell Death and Distinct Gene Expression

**DOI:** 10.1089/can.2018.0010

**Published:** 2018-06-01

**Authors:** Rameshprabu Nallathambi, Moran Mazuz, Dvory Namdar, Michal Shik, Diana Namintzer, Ajjampura C. Vinayaka, Aurel Ion, Adi Faigenboim, Ahmad Nasser, Ido Laish, Fred M. Konikoff, Hinanit Koltai

**Affiliations:** ^1^ Agricultural Research Organization, Volcani Center, Bet Dagan, Israel.; ^2^ The Mina and Everard Goodman Faculty of Life Sciences, Bar-Ilan University, Ramat Gan, Israel.; ^3^ The Interinstitutional Analytical Instrumentation Unit (IU), ARO, Volcani Center, Bet Dagan, Israel.; ^4^ Department of Gastroenterology and Hepatology, Meir Medical Center, Kfar Saba, Israel.; ^5^ Sackler Faculty of Medicine, Tel Aviv University, Tel Aviv, Israel.

**Keywords:** Cannabis, colorectal cancer, apoptosis, synergism, cell cycle arrest, cytotoxicity

## Abstract

**Introduction:** Colorectal cancer remains the third most common cancer diagnosis and fourth leading cause of cancer-related mortality worldwide. Purified cannabinoids have been reported to prevent proliferation, metastasis, and induce apoptosis in a variety of cancer cell types. However, the active compounds from *Cannabis sativa* flowers and their interactions remain elusive.

**Research Aim:** This study was aimed to specify the cytotoxic effect of *C. sativa*-derived extracts on colon cancer cells and adenomatous polyps by identification of active compound(s) and characterization of their interaction.

**Materials and Methods:** Ethanol extracts of *C. sativa* were analyzed by high-performance liquid chromatography and gas chromatograph/mass spectrometry and their cytotoxic activity was determined using alamarBlue-based assay (Resazurin) and tetrazolium dye-based assay (XTT) on cancer and normal colon cell lines and on dysplastic adenomatous polyp cells. Annexin V Assay and fluorescence-activated cell sorting (FACS) were used to determine apoptosis and cell cycle, and RNA sequencing was used to determine gene expression.

**Results:** The unheated cannabis extracts (C2F), fraction 7 (F7), and fraction 3 (F3) had cytotoxic activity on colon cancer cells, but reduced activity on normal colon cell lines. Moreover, synergistic interaction was found between F7 and F3 and the latter contains mainly cannabigerolic acid. The F7 and F7+F3 cytotoxic activity involved cell apoptosis and cell cycle arrest in S or G0/G1 phases, respectively. RNA profiling identified 2283 differentially expressed genes in F7+F3 treatment, among them genes related to the Wnt signaling pathway and apoptosis-related genes. Moreover, F7, F3, and F7+F3 treatments induced cell death of polyp cells.

**Conclusions:** *C. sativa* compounds interact synergistically for cytotoxic activity against colon cancer cells and induce cell cycle arrest, apoptotic cell death, and distinct gene expression. F3, F7, and F7+F3 are also active on adenomatous polyps, suggesting possible future therapeutic value.

## Introduction

Although there has been some reduction in mortality caused by colorectal cancer (CRC) due to advances in screening and preventive colonoscopies, it remains the third most common cancer diagnosis and fourth leading cause of cancer-related mortality worldwide.^1^ CRC is a heterogeneous disease that differs in clinical presentation, molecular characteristics, and prognosis.^2^ A series of histopathological and molecular changes lead the normal colonic epithelial cells to form aberrant crypt foci (ACF) and polyps that can further develop into CRC.^3^ As well, adenomatous polyps are recognized precursors of CRC.^4,5^ In addition to polypectomies, chemoprevention with natural or synthetic agents is another cornerstone of primary prophylactic intervention. Because the natural history of CRC is protracted, clinical trials have concentrated on preventing adenomas, which represent a form of intraepithelial neoplasia and are the precursors to carcinoma.

*Cannabis sativa* contains more than 500 constituents, among them more than a hundred terpenophenolic compounds termed phytocannabinoids.^6^ An increasing number of studies have shown that phytocannabinoids can prevent proliferation, metastasis, and angiogenesis, and induce apoptosis in a variety of cancer cell types, including breast, lung, prostate, skin, intestine, glioma, and others.^7^ This is due to their ability to regulate signaling pathways critical for cell growth and survival.^7^ Tetrahydrocannabinol (THC) treatment induced apoptosis in a CB1-dependent way in CRC cells and inhibited various survival signaling cascades while activating the proapoptotic BCL-2 family member BAD.^8^ Cannabidiol (CBD) reduced cell proliferation in colorectal carcinoma cell lines. In an animal model, it reduced ACF (preneoplastic lesions), polyps, and tumor formation and counteracted colon cancer-induced changes in gene expression.^9^ A CBD-rich cannabis extract also was shown to inhibit CRC cell proliferation and attenuate colon carcinogenesis.^10^ This activity involved both CB1 and CB2 receptor activation.^10^ Cannabigerol (CBG) promoted apoptosis, stimulated reactive oxygen species (ROS) production, and reduced cell growth in CRC cells. *In vivo*, CBG inhibited the growth of chemically induced colon carcinogenesis and xenograft tumors.^11^

Despite the accumulating knowledge on THC, CBD, and CBG, and receptor agonists or antagonists, only little is known on the other compounds in cannabis extracts that may have anticancer properties. Moreover, since advantages to the unrefined content of the inflorescence versus an isolated compound have been reported,^12,13^ beneficial interactions between active compounds should be examined.

In this study, we identified the *C. sativa* extract fractions and compounds that have cytotoxic activity on CRC cells and adenomatous polyps and evidenced their synergistic interaction. The interacting compounds induced cell cycle arrest, apoptotic cell death, and distinct gene expression.

## Materials and Methods

### Extraction of *Cannabis* inflorescence

Fresh inflorescences of *C. sativa* CS12 var were harvested from plants. They were either taken immediately for extraction and frozen at −80°C, or heated for 2.5 h at 150°C before extraction. Fresh and heated *Cannabis* inflorescences (2 g) were pulverized with liquid nitrogen. Absolute ethanol was added to each tube containing the powder at sample-to-absolute ethanol ratio of 1:4 (w/v). The tubes were mixed thoroughly on a shaker for 30 min and then the extract was filtered through a filter paper. The filtrate was transferred to new tubes. The solvent was evaporated with a vacuum evaporator. The dried extract was resuspended in 1 mL of absolute methanol and filtered through a 0.45-μm syringe filter (Merck, Darmstadt, Germany). For the treatments, the resuspended extract was diluted accordingly for cell cultures and biopsies in all experiments.

### Sample preparation

For high-performance liquid chromatography (HPLC), the dry extract was resuspended in 1 mL of methanol and filtered through a 0.45-μm syringe filter. The filtered extract was diluted 10 times with methanol and then separated by HPLC.

### HPLC separation and quantification

Sample separation was carried out in an UltiMate 3000 HPLC system coupled with WPS-3000(T) Autosampler, HPG-3400 pump, and DAD-300 detector. The separation was performed on a Purospher RP-18 endcapped column (250 mm×4.6 mm I.D.; Merck KGaA, Darmstadt, Germany) with a guard column (4 mm×4 mm I.D.). Solvent gradients were formed by isocratic proportion with 15% solvent A (0.1% acetic acid in water) and 85% solvent B (methanol) at a flow rate of 1.5 mL/min for 35 min. The compound peaks were detected at 220, and 280 nm. The 220-nm peaks were taken for further processing. The extracts were fractionated into nine fractions according to the obtained chromatogram. Tetrahydrocannabinolic acid (THCA; LGC standards) and cannabigerolic acid (CBGA; LGC standards) were used as external calibration standards for quantification of cannabinoids, at suitable concentrations ranging 5–20 μg.

### Gas chromatography coupled with mass spectrometer analysis

Gas chromatography (GC)/mass spectrometry (MS) analyses were carried out using a HP7890 GC coupled to a HP6973 mass spectrometer with electron multiplier potential 2 kV, filament current 0.35 mA, electron energy 70 eV, and the spectra were recorded over the range m/z 40 to 400. An Agilent 7683 autosampler was used for sample introduction. Helium was used as a carrier gas at a constant flow of 1.1 mL s^−1^. An isothermal hold at 50°C was kept for 2 min, followed by a heating gradient of 6°C min^−1^ to 300°C, with the final temperature held for 4 min. A 30 m, 0.25 mm I.D. 5% crosslinked phenylmethylsiloxane capillary column (HP-5MS) with a 0.25 μm film thickness was used for separation and the injection port temperature was 220°C. The MS interface temperature was 280°C. Peak assignments were performed with a spectral library (NIST 14.0) and compared with published and MS data obtained from the injection of standards (LGC standards). For identification and partial quantification, 5 μg of the most common cannabinoid standards, CBGA, cannabidiolic acid (CBDA), THCA, cannabichromene (CBC), and cannabinol (CBN), were dissolved in methanol and were injected to the GC/MS. Before GC/MS analysis, 200 μL of *N,O*-bis(trimethylsilyl)trifluoroacetamide (BSTFA; Sigma-Aldrich, St. Louis) containing 1% of trimethylchlorosilane (TMCS) was added to each completely dried extract and heated to 70°C for 20 min. One microliter of each sample was injected to the GC/MS using a 1:10 split ratio injection mode.

### Cell cultures

HCT 116 (ATCC CCL-247), HT-29 (ATCC HTB-38), Caco-2 (ATCC HTB-37), and CCD-18Co (ATCC CRL-1459) colon cells were grown at 37°C in a humidified 5% CO_2_–95% air atmosphere. Cells were maintained in McCoy's 5a Modified Medium (for HT-29 and HCT 116 cell lines), Dulbecco's Modified Eagle's Medium (DMEM; for Caco-2 cells) and Eagle's Minimum Essential Medium (EMEM; for CCD-18Co cell line; all cell lines were kindly provided by Prof. Margel, Bar Ilan University, Israel).

### Determination of extracts and compounds cytotoxic activity in cell lines

Resazurin (alamarBlue, R&D Systems, Minneapolis) was used to check the cytotoxic effect of extracts. For this, 10% Resazurin was added to each well of the treatments and incubated for 4 h at 37°C in a humidified 5% CO_2_–95% air atmosphere.

The relative fluorescence at the excitation/emission of 544/590 nm was measured. The percentage of live cells was calculated relative to the nontreated control after reducing the autofluorescence of alamarBlue without cells. Dose–effect curves of *C. sativa* ethanol extracts of fresh inflorescences (C2F), heated inflorescences (C2B) for HCT 116 colon cancer cells, and CCD-18Co colon healthy cells were determined. HCT 116 and CCD-18Co cells were seeded (10,000 per well) in triplicate in 100 μL growing media and incubated for 24 h at 37°C in a humidified 5% CO_2_–95% air atmosphere. Cells were treated with C2F, C2B at different dilutions (35–1600 μg/mL) along with 50 ng/mL tumor necrosis factor (TNF-α) for 16 h. Afterward, the viability of the cells was determined with alamarBlue. GraphPad Prism was employed to produce a dose–response curve and IC50 doses of C2F and C2B.

### XTT viability assay

Cells were seeded into a 96-well plates at a concentration of 10,000 cells per well in triplicate in normal growing media. The following day, the media were replaced with normal growing media containing plant extracts/fractions, standards (CBGA and THCA), or media only for control (as mentioned in each experiment). Cells were incubated for 48 h, after which XTT (2,3,-bis(2-methoxy-4-nitro-5-sulfophenyl)-5-[(phenylamino)-carbonyl]-2H-tetrazolium inner salt) reduction was used to quantify viability according to the manufacturer's instruction (BI, Kibbutz Beit-Haemek, Israel). Cells were incubated with XTT reagent for 2 h at 37°C in a humidified 5% CO_2_–95% air atmosphere. Absorbance was recorded by a photometer, SPEKTRA Fluor Plus (Tecan, Salzburg, Austria), at 490 nm with 650 nm of reference wavelength. Cell survival was estimated from the equation: % cell survival=100×(At − Ac)(treatment)/(At − Ac)(control); At and Ac are the absorbencies (490 nm) of the XTT colorimetric reaction (BI) in treated and control cultures, respectively, minus nonspecific absorption that was measured at 650 nm. Absorbance of medium alone was also deducted from specific readings.

### Analysis of combined effects

To examine synergy between F3 and F7 cytotoxic activity, XTT assay was used on HCT 116 cells as described above. Different concentrations of F3 (3.7–80.0 μg/mL), with and without the IC50 dose of F7 (20 μg/mL), or different concentrations of F7 (7.9–63.0 μg/mL) with and without the IC50 dose of F3 (36 μg/mL) were used to treat the cells for 48 h. Next, the cells were incubated with XTT reagent for 2 h as described above. For examination of synergy between standards, different concentrations of THCA (4.0–50.0 μg/mL) with and without CBGA (28 μg/mL), or different concentrations of CBGA (6.7–53.3 μg/mL) with and without THCA (13.14 μg/mL) were used. The range of concentrations for examination of synergy on cell viability for the THCA or CBGA standards was determined based on quantification of THCA in F7 or CBGA in F3 using HPLC (as described above). Drug synergy was determined by Bliss independence drug interaction model,^14^ which is defined by the following equation:

\begin{align*}
{{ \rm{E}}_{{ \rm{xy}}}} = {{ \rm{E}}_{ \rm{x}}} + {{ \rm{E}}_{ \rm{y}}} - \left( {{{ \rm{E}}_{ \rm{x}}}{{ \rm{E}}_{ \rm{y}}}} \right) ,
\end{align*}

where (E_xy_) is the additive effect of the drugs x and y as predicted by their individual effects (E_x_ and E_y_). For the calculation purposes in this article, the anticancer effect of the drug was defined as complementary to the obtained results (1−E_xy_). In case the observed value of E_xy_ is greater than the calculated E_xy_ value, the combination treatment is considered antagonistic. If the observed value is less than the calculated one, then the combination treatment is considered synergistic. If both values are equal, the combination treatment is considered additive (independent).

Drug synergy was also determined by combination index (CI) methods, derived from the median-effect principle.^15^ Data obtained from the growth inhibitory experiments were used to perform these analyses. Combination data points that fall on the line represent an additive drug–drug interaction, whereas data points that fall below or above the line represent synergism or antagonism, respectively. The CI method is a mathematical and quantitative representation of a two-drug pharmacologic interaction. Using data from the growth inhibitory experiments, CI value was calculated using CompuSyn software (ComboSyn, Inc.) as described in the equation below

\begin{align*}
 { \rm { CI = } } { \frac { { { \rm { C } } _ { { \rm { A , x } } } } }  { { \rm { I } } { { \rm { C } } _ { { \rm { x , \;A } } } } { \rm { } } } } + { \frac { { { \rm { C } } _ { { \rm { B , x } } } } }  { { \rm { I } } { { \rm { C } } _ { { \rm { x , B } } } } } } 
\end{align*}

C_A,x_ and C_B,x_ are the concentrations of drug A and drug B used in combination to achieve percentage of drug effect. IC_x,A_ and IC_x,B_ are the concentrations for single agents to achieve the same effect. CI values are generated over a range of fraction affected levels from 0.25 to 0.90 (25%–90% growth inhibition). A CI of 1 indicates an additive effect between two drugs, whereas a CI <1 or CI >1 indicates synergism or antagonism, respectively.

### Annexin V assay

Apoptosis was assessed using the MEBCYTO Apoptosis Kit with Annexin V-FITC and Propidium Iodide (PI) (MBL, Enco, 4700). Staining was done according to the manufacturer's instructions. In brief, cells were seeded in 6-well plate culture dishes, at density of 1×10^6^ cells per well in McCoy's 5a Modified Medium. The following day, the medium was replaced with medium containing IC-50 dose of F7 (20 μg/mL), F3 (35 μg/mL) and combination of F7 and F3 along with TNF-α (50 ng/mL) and incubated for 24 and 48 h at 37°C in a humidified 5% CO_2_–95% air atmosphere. After incubation, cells were harvested and collected separately. Then tubes were centrifuged for 10 min at 900 *g* relative centrifugal force (RCF) and cell pellets were resuspended and washed twice with 1 mL of phosphate-buffered saline (PBS). The cells in each sample were counted and if necessary, the number of cells was adjusted to a concentration of 2×10^5^ cells in 85 μL of Annexin binding buffer. Cells were stained using 10 μL of Annexin V-FITC solution and 5 μL of PI working solution followed by incubation in darkness at room temperature for 15 min. Then 400 μL of Annexin V binding buffer was added to each tube and flow cytometry was performed using GALLIOS flow cytometer (fluorescence-activated cell sorting [FACS]). Cells were considered to be apoptotic if they were Annexin V+/PI- (early apoptotic) and Annexin V+/PI+ (late apoptotic). Live cells were Annexin V-/PI- and Annexin V-/PI+ are the necrosis.

### Cell cycle analysis

Cells were seeded in 6-well plate culture dishes at a density of 1×10^6^ cells per well. After 24 h of seeding, the cell culture media were replaced with starvation media and incubated for 24 h at 37°C in a humidified 5% CO_2_–95% air atmosphere. After 24 h of incubation, the cells were treated with F7 (20 μg/mL), F3 (36 μg/mL), F7 in combination with F3 and solvent control along with TNF-α (50 ng/mL) for another 24 h. Then the cells from each well were harvested and collected separately and centrifuged for 10 min at 900 *g*. The cell pellets were washed once with 1 mL of PBS and fixed with 70% cold ethanol at 4°C for 1 h. The fixed cells were then pelleted out and washed twice with 1 mL of PBS. The cell pellet was then stained by resuspending in 250 μL of PI solution (50 μg/mL) containing RNase A (100 μg/mL) for 15 min in darkness. Then 400 μL of PBS was added to each tube and the cells were analyzed using GALLIOS flow cytometer.

### Culture of biopsies

Biopsies from polyps and healthy colonic tissue from the same patient were obtained from seven patients scheduled for colonoscopies deemed necessary by their physicians. The study was approved by our Institutional Ethics Committee (Helsinki approval no. 0121-16), and all patients gave their written informed consent before the colonoscopy. Biopsies taken during each colonoscopy were placed in tissue culture media and immediately transported to the laboratory. Upon receiving the biopsies, the PBS was replaced with Hank's Balanced Salt Solution and the samples were centrifuged at 8000 rpm (5,939 *g*) for 1 min. The supernatant was then removed and the tissues were washed four times with Hank's Balanced Salt Solution. After each wash, samples were centrifuged as described above. The tissues were placed in a small Petri dish and cut into 4–5 pieces with a clean scalpel. The pieces were then placed on Millicell hydrophilic PTFE tissue-culture inserts (30 mm, 0.4 μm; Millipore). The inserts were placed in 6-well plastic tissue culture dishes (Costar 3506) along with 1.5 mL of tissue culture medium (DMEM supplemented with 10% v/v heat-inactivated fetal calf serum, 100 U/mL penicillin, 100 μg/mL streptomycin, 50 μg/mL leupeptin, 1 mM PMSF, and 50 μg/mL soybean trypsin inhibitor). This treatment was followed by treating the tissues with extracts, or leaving them untreated (control). The treatment medium was replaced with a medium containing C2F (1.25 mg/mL), F7 (at different concentrations: 100, 125, 250, or 400 μg/mL), F3 (75, 107, or 176 μg/mL), or F3+F7 (at the desired concentrations) and incubated overnight at 37°C in a humidified 5% CO_2_–95% air atmosphere.

### Cell separation and Resazurin for biopsies

After 16 h, the treated and untreated tissues from the above section were taken into a tube and washed twice with PBS. Then the tissues were transferred into a Petri dish and chopped into very fine pieces using a surgical scalpel. The finely chopped pieces were transferred into tubes and 500 μL of R10 medium (RPMI 1640 supplemented with 10% FBS, 10 mM HEPES, 100 U/mL penicillin, 100 μg/mL streptomycin, and 50 μg/mL gentamicin) was added along with 20 IU/mL of DNase, 0.13 units/mL of dispase, and 1 mg/mL of collagenase 1A. Then the tissues were vortexed and incubated at 37°C for 1 h by vortexing every 15 minutes in between. Subsequently, the cell suspensions were pelleted at 950 *g* for 10 min and washed twice with PBS buffer. The cell suspension pellets were resuspended with 500 μL R10 medium and incubated at 37°C in a humidified 5% CO_2_–95% air atmosphere with 10% Resazurin for 4 h. The supernatant (100 μL from each well) was transferred to a 96-well plate and the relative fluorescence at the excitation/emission of 544/590 nm was measured. The percentage of live cells was calculated relative to the nontreated control after reducing the autofluorescence of alamarBlue without cells.

### RNA sequencing and transcriptome analysis

For RNA preparation, cells were seeded into a 6-well plate at a concentration of 1,500,000 cell/mL per well. After 24 h of incubation at 37°C in a humidified 5% CO_2_–95% air atmosphere, cells were treated with F3 (36 μg/mL), F7 (20 μg/mL) and combination of F3 with F7 at these concentrations along with TNF-α (50 ng/mL) for 6 h. The cells were next harvested and total RNA was extracted using a TRI reagent (Sigma-Aldrich) according to the manufacturer's protocol. The RNA was kept at −80°C until further analysis. Sequencing libraries were prepared using the INCPM mRNA Seq protocol. Sixty base pair single reads were sequenced on 1 lanes of an Illumina HiSeq.

For transcriptome analysis, the raw-reads were subjected to a filtering and cleaning procedure. The SortMeRNA tool was used to filter out rRNA.^16^ Next, the FASTX Toolkit (http://hannonlab.cshl.edu/fastx_toolkit/index.html, version 0.0.13.2) was used to trim read-end nucleotides with quality scores <30, using the FASTQ Quality Trimmer, and to remove reads with less than 70% base pairs with a quality score ≤30 using the FASTQ Quality Filter.

Clean reads were aligned to the human genome extracted from National Center for Biotechnology Information (NCBI) (GRCh38; https://www..ncbi.nlm.nih.gov/genome/guide/human/) using Tophat2 software (v2.1).^17^ Gene abundance estimation was performed using Cufflinks (v2.2)^18^ combined with gene annotations from the NCBI. Heatmap visualization was performed using R Bioconductor.^19^ Differential expression analysis was completed using the edgeR R package.^20^ Genes that varied from the control more than twofold, with an adjusted *p*-value of no more than 0.05, were considered differentially expressed.^21^ Venn diagrams were generated using the online tool at bioinformatics.psb.ugent.be/webtools/Venn/. Functional annotation of the significant expressed genes was extended using PANTHER (www.pantherdb.org/), based on gene ontology (GO) categories assigned to the human. The KEAGG database (www.genome.jp/kegg/) was used for pathways analysis using the KEAGG mapper tool (www.genome.jp/kegg/tool/map_pathway2.html).

### Statistical analyses

Results are presented as mean±SE of replicate analyses and are either representative of or include at least two independent experiments. Mean of replicates was subjected to statistical analyses by Tukey–Kramer test (*p*≤0.05) using the JMP statistical package and considered significant when *p*≤0.05. Different letters above bars indicate statistically significant differences between mean by one-way analysis of variance (ANOVA). For dose–response assays, data points were connected by nonlinear regression lines of the sigmoidal dose–response relation. GraphPad Prism was employed to produce dose–response curve and IC50 doses. FlowJo software was used to analyze FACS data.

## Results

### *C. sativa* extracts from fresh inflorescences are active in reducing cell viability in colon cancer cell lines

**FIG. 1.**
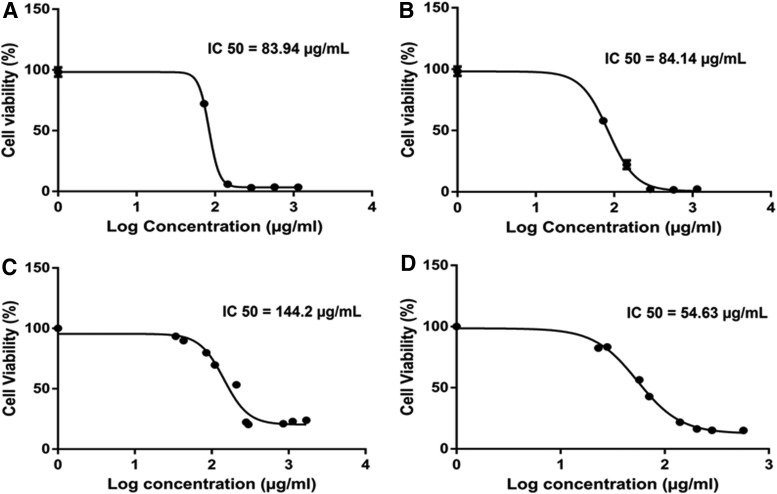
Dose–effect curves of *Cannabis sativa* ethanol extracts on the viability of HCT 116 colon cancer and CCD-18Co colon healthy cells. Dose–effect curves of *C. sativa* ethanol extracts of fresh inflorescences (C2F) (**A**; IC50=83.9±0.9 μg/mL, 95% confidence interval=83.2–84.7), heated inflorescences (C2B) (**B**; IC50=84.1±1.3 μg/mL, 95% confidence interval=83.1–85.2) on the viability of HCT 116 colon cancer cells, and C2F (**C**; IC50=144.2±1.1 μg/mL, 95% confidence interval=142.9–145.5), C2B (**D**; IC50=54.63±2.03 μg/mL, 95% confidence interval=53.09–56.17) on the viability of CCD-18Co colon healthy cells. Cell viability determined by alamarBlue fluorescence (Resazurin assay). HCT 116 and CCD-18Co cells were seeded (10,000 per well) in triplicate in 100 μL of growing media and incubated for 24 h at 37°C in a humidified 5% CO_2_-95% air atmosphere. Cells were treated with C2F, C2B at different dilutions along with 50 ng/mL TNF-α for 16 h. The cells were next incubated with alamarBlue for 4 h. Relative fluorescence at the excitation/emission of 544/590 nm was measured. Values were calculated as the percentage of live cells relative to the nontreated control (cells without TNF-α and treatments) after reducing the autofluorescence of alamarBlue without cells (*n*=3). For dose–response assays, data points were connected by nonlinear regression lines of the sigmoidal dose–response relation. GraphPad Prism was used to produce dose–response curve and IC50 doses for C2F and C2B. TNF, tumor necrosis factor.

Cytotoxic activity was determined as the level of cell viability in HCT 116 cells for absolute ethanol extracts of fresh (C2F) and heated (C2B) inflorescences of *C. sativa* (CS12 var.) following overnight treatment. Treatments with C2F or C2B were found to significantly reduce HCT 116 cancer cell viability with a similar level of activity (Fig. 1A, B). Moreover, although both ethanol extracts of *C. sativa*, C2F and C2B, have similar cytotoxic activity on colon cancer cells HCT 116 (Fig. 1A, B), the activity of C2F on CCD-18Co healthy colon cells was reduced. C2F had IC50 of 83.9 and 144.2 μg/mL on HCT 116 and CCD-18Co cell lines, respectively (Fig. 1A, C). On the other hand, C2B was more active on CCD-18Co than on HCT 116 cell lines, with IC50 of 54.63 and 84.1 μg/mL, respectively (Fig. 1B, D).

We previously determined the chemical composition of *C. sativa* extracts from fresh and heated inflorescences. CBD, CBG, and THC were found in C2B, whereas in C2F the acidic forms of all the above compounds (i.e., CBDA, CBGA, THCA) were mostly present.^22^

### C2F and F7 have cytotoxic activity on human colon polyp biopsies

Adenomatous polyps are the primary premalignant precursors of CRC. Hence, to examine a possibility for a therapeutic or preventive potential of the extracts, we studied biopsies of adenomatous polyps and healthy tissue from patients scheduled for colonoscopy. Biopsy tissues of polyps and normal colon tissue of the same patient were exposed to C2F and F7 for 16 h followed by cell separation and Resazurin assay to determine tissue cell viability. Both C2F and F7 treatments significantly reduced cell viability of both polyp and healthy tissues (Table 1).

**Table 1.** ***Cannabis sativa* C2F and F7 Cytotoxic Activity on Human Colon Polyp and Healthy Colon Tissue**

Sample	Treatment	% Living cells	Statistics
Healthy	NT+MeOH	100	A
	C2F (1.25 mg/mL)	18.41	B
	F7 (0.4 mg/mL)	18.43	B
Polyp	NT+MeOH	100	a
	C2F (1.25 mg/mL)	7.81	b
	F7 (0.4 mg/mL)	13.30	b

Cytotoxic activity was calculated as % of living cells from control of tissue treated with methanol only (NT+MeOH). Healthy biopsy of normal tissue, *n*=3; polyp biopsy of adenomatous polyp, *n*=3.

NT, nontreated.

### F7 interaction with other *C. sativa* cannabis fractions induces cytotoxic activity

C2F is as active as C2B on cancer cells, but is less active on normal cell lines; therefore, C2F was further analyzed for cytotoxic activity. Previously, we showed that the HPLC fraction 7 of C2F F7 (that contains mainly THCA) has only moderate cytotoxic activity against HCT 116. However, the combination of F7 with the other C2F fractions led to a marked increase in cytotoxic activity.^22^ In this case, the interaction between F7 and the other *C. sativa* C2F fractions was further examined for each fraction separately.

**FIG. 2.**
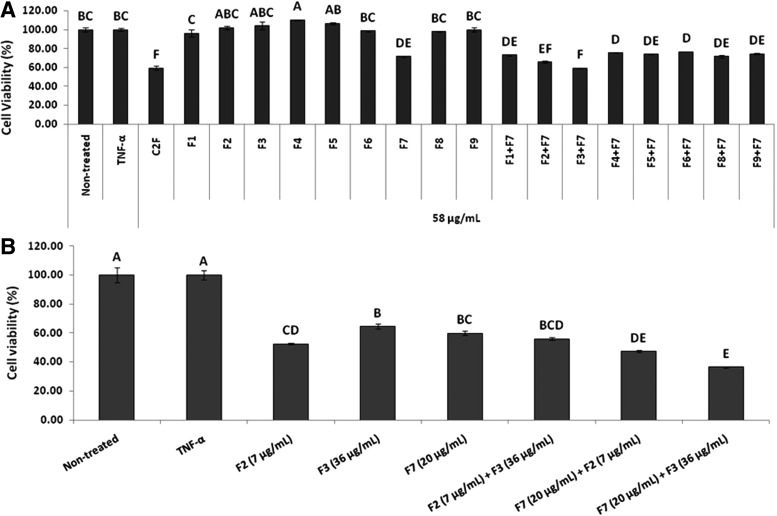
Effect of *Cannabis sativa* C2F and HPLC fractions (F1–F9) in different combinations on HCT 116 cell viability. **(A)** Determination of HCT 116 cell viability using XTT assay as a function of live cell number. Cells were seeded and treated with *C. sativa* ethanol extracts (C2F) F1–F9, excluding F7 (HPLC fractions of C2F) and F1–F9, including F7 (HPLC fractions of C2F) at the IC50 dose of C2F crude (58 μg/mL), and F1–F9 diluted as C2F crude along with 50 ng/mL of TNF-α for 48 h. The cells were then incubated with XTT reagent for 2 h. Absorbance was recorded at 490 nm with 650 nm of reference wavelength. Values were calculated as the percentage of live cells relative to the nontreated (cells without TNF-α and treatments) control after reducing the absorbance without cells. **(B)** Determination of synergism of C2F Fraction 7 (F7) in combination with Fraction 2 (F2) and Fraction 3 (F3) on HCT 116 cell viability using XTT assay as a function of live cell number. Cells were seeded and treated with IC50 doses of F2 (7 μg/mL), F3 (36 μg/mL), F7 (20 μg/mL), the combinations of F2+F3, F7+F2, and F7+F3, along with 50 ng/mL of TNF-α for 48 h. Subsequently, the cells were incubated with XTT reagent for 2 h. Absorbance was recorded at 490 nm with 650 nm of reference wavelength. Values were calculated as the percentage of live cells relative to the nontreated control (cells without TNF-α and treatments) after reducing the absorbance without cells. Error bars indicate ±SE (*n*=3). Levels with different letters are significantly different from all combinations of pairs by Tukey–Kramer HSD. HPLC, high-performance liquid chromatography; HSD, honest significant difference.

Forty-eight hour treatment with F7 led to only a moderate effect on cell viability, whereas combinations of F7 with F2 or F3 was found to have increased cytotoxic activity (Fig. 2A). Only combination of F7 and F3 (in concentrations found in C2F) resulted with increased cytotoxic activity despite the low (F3) to moderate (F7) activity of each (Fig. 2B). Both F7 and F7+F3 treatments were cytotoxic to HT-29 and Caco-2 cell lines (Supplementary Fig. S1). As expected, F7+F2 or F7+F3 were much less potent on the normal CCD-18Co cell line (Supplementary Fig. S2).

### Synergistic interaction of *C. sativa* fractions F7+F3 and F3 chemical composition

To determine whether the interaction of F7 and F3 is synergistic, that is, their combined activity is greater than the sum of their separate activities, the extent of activity in different combined concentrations of F7 and F3 was examined. The IC50 of F7 and F3 was determined to be 21.7 and 35.47 μg/mL, respectively (Supplementary Fig. S3). Subsequently, the partial effect of the drugs was calculated according to the Bliss Independence Model for each combination experiment. Four to six concentrations of each combination were examined. Synergistic interactions were found for the following combinations: F7 at its IC50 (21.7 μg/mL)+F3 at concentrations of 26.7, 20.0, and 13.3 μg/mL; and F3 at its IC50 (35.5 μg/mL)+F7 at concentrations of 15.8, 11.9, and 7.9 μg/mL (Table 2). Since the Bliss Independence Model has high risks on false-positive results,^23^ we have confirmed synergy between F7 and F3 by the CI method (Supplementary Table S1).

**Table 2.** **Synergism Calculation for Combinations of Fractions (F7 and F3)**

	A. F7 (20 μg/mL)					
	F3 (40.0 μg/mL)	*F3 (26.7* μ*g/mL)*	*F3 (20* μ*g/mL)*	*F3 (13.3* μ*g/mL)*	F3 (10 μg/mL)	F3 (6.7 μg/mL)
Calculated value	22.64	50.42	52.13	52.45	52.52	54.49
Experimental value	32.21	38.94	43.31	51.86	59.61	69.24

	B. F3 (36 μg/mL)			
	F7 (23.8 μg/mL)	*F7 (15.8* μ*g/mL)*	*F7 (11.9* μ*g/mL)*	*F7 (7.9* μ*g/mL)*
Calculated value	29.95	49.84	58.67	58.06
Experimental value	32.16	33.96	38.85	41.97

(A) F7 in constant concentration of 20 μg/mL and F3 in different concentrations (6.7–40 μg/mL). Italicized values are concentrations, which showed synergism between fractions, as was determined by XTT assay on HCT 116 cells. (B) F3 in constant concentration of 36 μg/mL and F7 in different concentrations (7.9–23.8 μg/mL). Italicized values are concentrations, which showed synergism between fractions, as was determined by XTT assay on HCT 116 cells. The partial effect of the drugs was calculated according to the Bliss Independence Model for each combination experiment.

Furthermore, the combination of F3 at its IC50 with F7 resulted in approximately a threefold reduction in F3 IC50 (from 35.5 to 10.8 μg/mL). The combination of F7 at its IC50 with F3 resulted in an 11-fold reduction in F7 IC50 (from 21.7 to 1.9 μg/mL).

F3 was found to contain mainly CBGA (at 91.20%), CBN (3.67%), CBCA (3.53%), terpenes and terpene-ethanol compounds (0.72%), diterpenes (0.33%), and short free fatty acids (0.37%). The rest (0.18%) of compounds present in F3 are unidentified. Combined treatments with the purified compounds that constitute most of the fractions, that is, THCA in F7 and CBGA in F3, also resulted in synergistic interactions (Table 3). We have confirmed synergy between THCA and CBGA by the CI method (Supplementary Table S2).

**Table 3.** **Synergism Calculation for Combinations of Standards (THCA and CBGA)**

	A. THCA (13.1 μg/mL)					
	CBGA (53.3 μg/mL)	*CBGA (40.0* μ*g/mL)*	*CBGA (28.0* μ*g/mL)*	*CBGA (20.0* μ*g/mL)*	*CBGA (13.3* μ*g/mL)*	*CBGA (6.7* μ*g/mL)*
Calculated value	15.10	17.69	22.97	50.19	44.37	52.49
Experimental value	15.69	15.15	16.89	16.86	19.29	19.44

	B. CBGA (28.0 μg/mL)						
	THCA (50.0 μg/mL)	*THCA (30.0* μ*g/mL)*	*THCA (25.0* μ*g/mL)*	*THCA (15.0* μ*g/mL)*	*THCA (12.0* μ*g/mL)*	*THCA (6.0* μ*g/mL)*	*THCA (4.0* μ*g/mL)*
Calculated value	10.53	20.43	21.25	44.66	47.60	70.59	69.18
Experimental value	16.56	14.40	16.90	23.25	28.69	29.68	42.44

(A) THCA in constant concentration of 13.1 μg/mL and CBGA in different concentrations (6.7–53.3 μg/mL). Italicized values are concentrations, which showed synergism between standards, as was determined by XTT assay on HCT 116 cells. (B) CBGA in constant concentration of 28 μg/mL and THCA in different concentrations (4.0–50.0 μg/mL). Italicized values are concentrations, which showed synergism between standards, as was determined by XTT assay on HCT 116 cells. The partial effect of the drugs was calculated according to the Bliss Independence Model for each combination experiment.

CBGA, cannabigerolic acid; THCA, tetrahydrocannabinolic acid.

### Treatment of HCT 116 cells with *C. sativa* F7 and F7+F3 induced apoptotic cell death

**FIG. 3.**
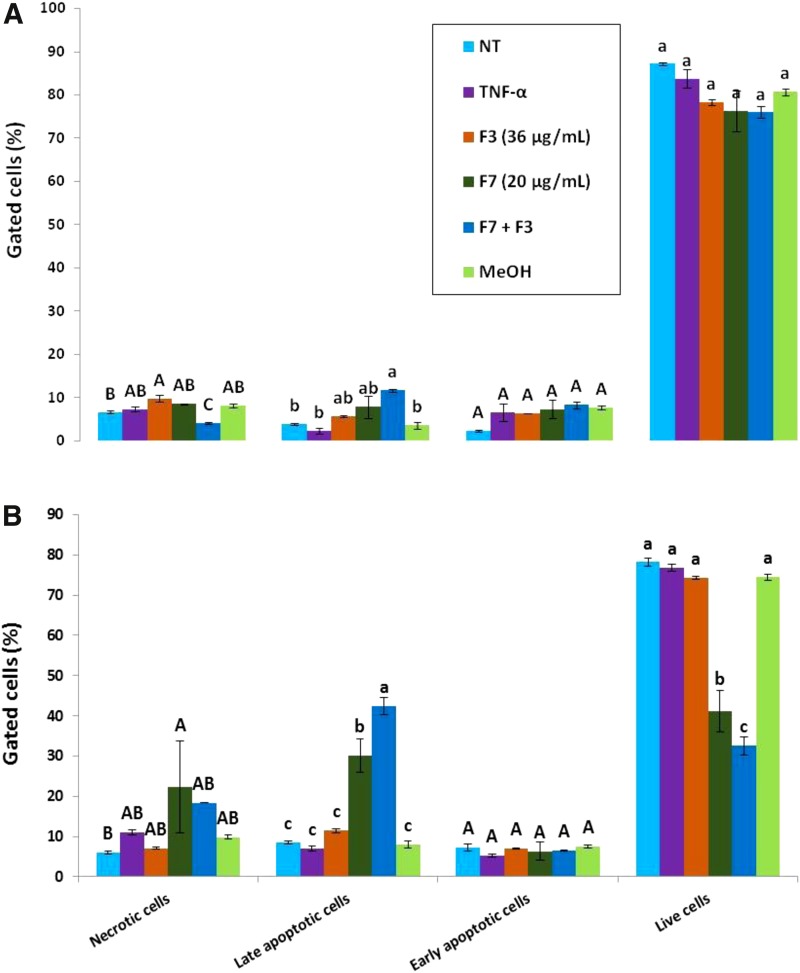
Determination of apoptosis or necrosis as cytotoxic effect of F7, F3, or F7+F3 on HCT 116 cells. HCT 116 cells were treated with F7 (20 μg/mL), F3 (36 μg/mL), the combination of F7 with F3 and solvent control (methanol) along with TNF-α (50 ng/mL) for 24 h **(A)** or 48 h **(B)**. The treated cells were harvested and analyzed in FACS following Annexin V-FITC and PI staining. Shown are the percentages of live, necrotic, early, and late apoptosis cells, analyzed from 10,000 events per treatment. FACS, fluorescence-activated cell sorting; PI, propidium iodide.

Cell sorting for cell viability by FACS based on Alexa Fluor^®^ 488/Annexin V staining suggested that treatment for 48 h with F7 leads to a large proportion of cells that are in early or late apoptosis in comparison to controls (nontreated and TNF-α or Methanol [MeOH]-treated cells); for example, 8.5±0.4 or 30.1±4.1 for nontreated (NT) or F7 for late apoptosis, respectively. This proportion was even significantly higher in F7+F3-treated cells at 48 h (42.5±2.1 for late apoptosis), but treatment with F3 only did not lead to cell death (Fig. 3 and Supplementary Fig. S4). At 24 h, apoptosis was not yet evident, but a slight and significant reduction of cell necrosis was found with the F7+F3 treatment (4.0±0.2 or 6.7±0.3 for F7+F3 or NT, respectively; Fig. 3 and Supplementary Fig. S4). These results suggest that F7 or F7+F3 treatments may act through induction of apoptosis.

### Treatment of HCT 116 cell line with *C. sativa* F7 or F7+F3 leads to S or G0/G1 cell cycle arrest, respectively

**FIG. 4.**
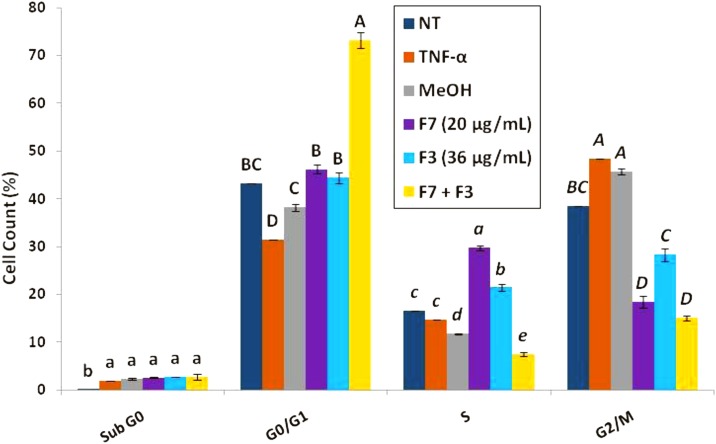
Determination of stages of cell cycle arrest induced by F7, F3, or F7+F3 in HCT 116 cells. Starved HCT 116 cells were treated with F7 (20 μg/mL), F3 (36 μg/mL), the combination of F7 with F3 and solvent control (methanol) along with TNF-α (50 ng/mL) for 24 h. The treated cells were harvested, fixed, and analyzed in FACS following PI staining. The percentage of cells in Sub-G0, G0/G1, S, and G2/M phase were analyzed from 10,000 events per treatment.

Cell sorting for cell cycle analysis by FACS based on PI staining suggested that treatment of the cells with F7+F3 led at 24 h to a marked increase in proportion of cells in G0/G1 phase in comparison to controls (NT and treated with TNF-α or MeOH; 43.2±0.0 or 73.2±1.7 for NT or F7+F3, respectively; Fig. 4) Treatment with F7 or F3 (F3 to a lesser extent) led to an increase in cells in S phase in comparison to controls (Fig. 4).

### F7 and F7+F3 have cytotoxic activity on human colon polyp biopsies

We next examined whether treatment with F7+F3 leads to an increase in the cytotoxic activity. We treated biopsy tissues of polyps and normal colon tissue from the same patient with reduced concentrations of F7, F3, or F7+F3 for 16 h followed by cell separation and Resazurin assay to determine tissue cell viability. Results varied between patients (*n*=4). For some, F7+F3 treatment was more effective than only F7 or F3, whereas for other patients, treatment with F7+F3 did not improve cytotoxicity in comparison to F7 or F3 only (Table 4). In all cases but one (P4), treatments reduced polyp cell viability.

**Table 4.** ***Cannabis sativa* F7, F3, and F7+F3 Cytotoxic Activity on Human Colon Polyp and Healthy Colon Tissue**

		% Living cells			
Sample		P1	P2	P3	P4
Healthy tissue	NT	100^A^	100^A^	100^**A**^	100^*B*^
	F3	68.5^B^	51.3^B^	18.1^**B**^	122.5^*A*^
	F7	54.4^C^	25.2^C^	4.9^**C**^	97.3^*C*^
	F3+F7	55.4^BC^	30.0^C^	7.6^**C**^	16.3^*D*^
Polyp	NT	100^a^	100^a^	100^**a**^	100^c^
	F3	24.4^b^	77.2^b^	13.0^**c**^	119.7^b^
	F7	15.4^c^	63.4^bc^	5.8^**d**^	142.1^a^
	F3+F7	16.1^c^	51.7^c^	33.5^**b**^	40.6^d^

Cytotoxic activity was calculated as % of living cells from control of tissue treated with methanol only (NT+MeOH). Healthy biopsy of normal tissue (*n*=4); Polyp biopsy of adenomatous polyp (*n*=4). Percentages with different letters are significantly different from all combinations of pairs by Tukey HSD.

HSD, honest significant difference.

### F7+F3 treatment induces distinct profile of gene expression in HCT 116 cells in comparison to F7 or F3 treatments

**FIG. 5.**
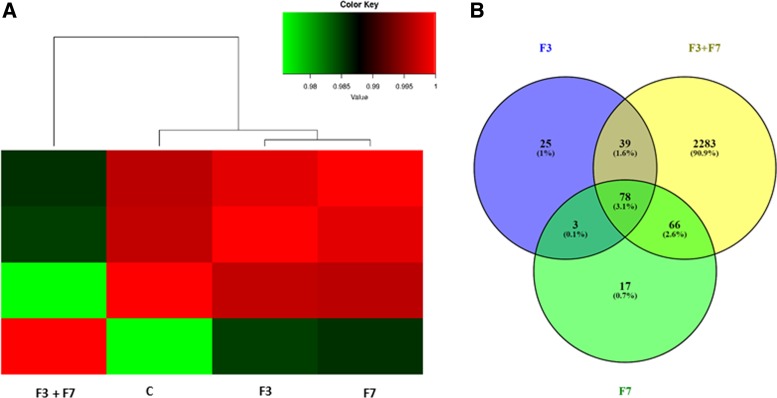
Hierarchical clustering and Venn diagram of genes significantly differentially expressed in HCT 116 cells treated with F7, F3, or F7+F3. **(A)** Hierarchical clustering and Pearson correlations among the four conditions based on the gene expression (counts-per-million) followed by a log2 transform. Pearson correlations were calculated with the R software. **(B)** Venn diagrams illustrating the relationships between significantly differentially expressed genes in the three treatments against the control.

To identify genes differentially expressed in HCT 116 cells following treatment with *C. sativa* extract fractions, we performed RNA sequence analysis of HCT 116 cells 6 h post-treatment with F7, F3, or F7+F3. Sample correlation tests of RNA sequencing results suggested that those of the cells treated with F7 or F3 were clustered together, and those of control treatment clustered in a separate clade. However, RNA sequencing results of the treatment with F7+F3 at the concentrations shown above to act synergistically (i.e., 20 mg/mL for F7 and 36 μg/mL for F3) were clustered as an outgroup clade to the rest of the treatments (Fig. 5A).

**FIG. 6.**
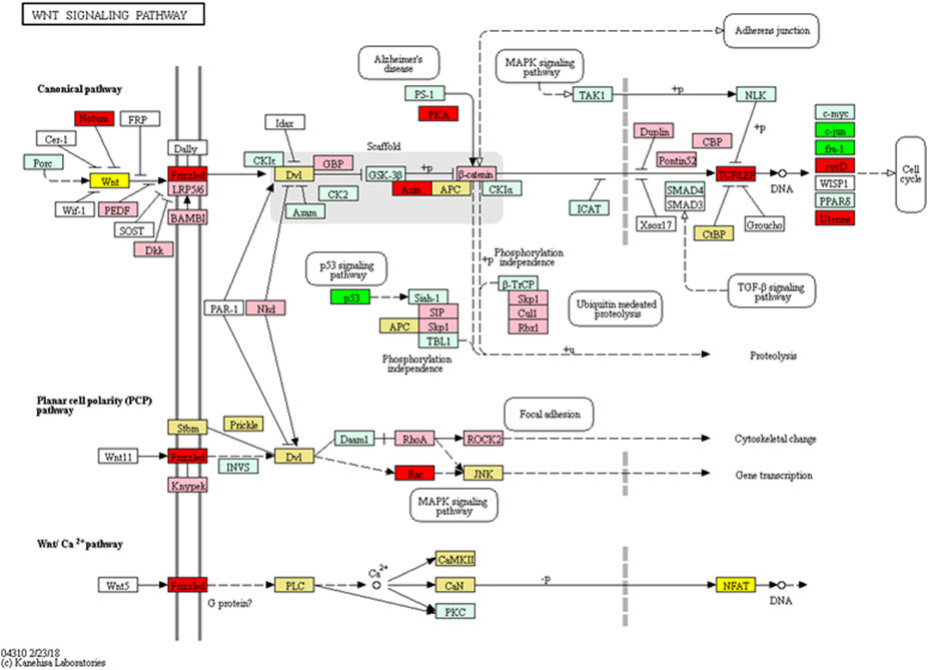
Genetic pathways of genes differentially expressed in HCT 116 cells treated with F7+F3 versus control for Wnt signaling pathways. Pathways determined according to KEGG (www.genome.jp/kegg/). Green boxes—significantly upregulated genes; red boxes—significantly downregulated genes (edgeR; more than twofold and *padj* <0.05). Light green boxes—nonsignificant upregulated genes; pink boxes—nonsignificant downregulated genes. Yellow boxes denote genes with multiple gene annotations that encompass both significantly upregulated and downregulated genes; light yellow boxes denote genes with multiple gene annotations that encompass both nonsignificant upregulated and nonsignificant downregulated genes.

**FIG. 7.**
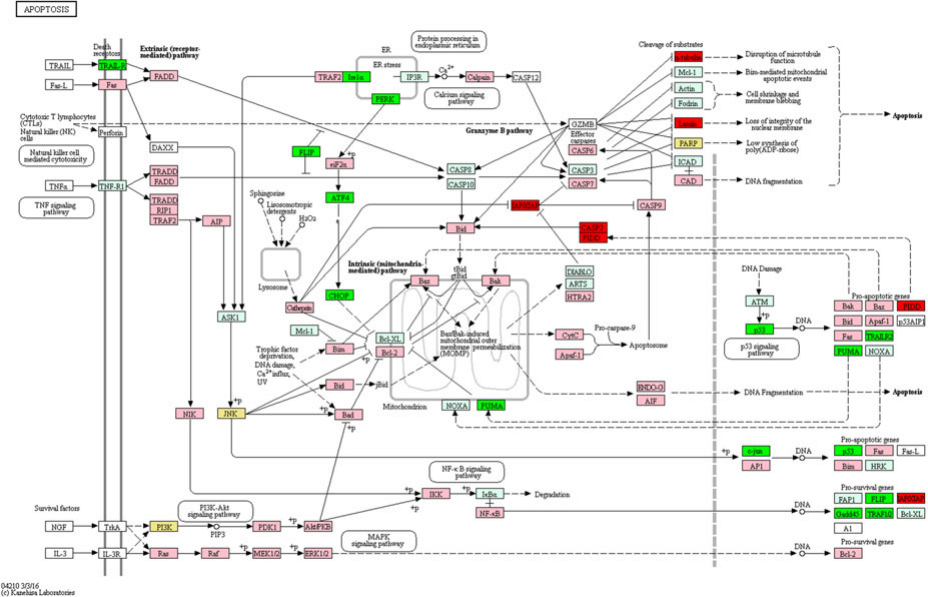
Genetic pathways of genes differentially expressed in HCT 116 cells treated with F7+F3 versus control for apoptotic signaling pathways. Pathways determined according to KEGG (www.genome.jp/kegg/). Green boxes—significantly upregulated genes; red boxes—significantly downregulated genes (edgeR; more than twofold and *padj* <0.05). Light green boxes—nonsignificant upregulated genes; pink boxes—nonsignificant downregulated genes. Yellow boxes denote genes with multiple gene annotations that encompass both significantly upregulated and downregulated genes; light yellow boxes denote genes with multiple gene annotations that encompass both nonsignificant upregulated and nonsignificant downregulated genes.

In the experiments, 2283 genes were found to be differentially expressed in cells treated with F7+F3, but not in cells treated with F7 or F3 only, compared with the control (Fig. 5B). Among the differentially expressed genes specific to the F7+F3 treatment to be discussed in this study are those involved with cell cycle G1/S phase transition, Wnt signaling pathway (Fig. 6), and p53 and apoptosis signaling pathways (Fig. 7). Gene expression data of those genes are listed in Supplementary Table S3.

## Discussion

The present study provides evidence of a synergistic interaction of cytotoxic activity against colon cancer cells between two *C. sativa* extract fractions. F7 contains mainly THCA,^22^ whereas F3 contains mainly CBGA but with additional minute amounts of other cannabinoids and terpenes. Several cell-based experiments have demonstrated that THCA has immunomodulatory, anti-inflammatory, neuroprotective, and antineoplastic activity.^24^ CBGA was shown to have cytotoxic activity with IC50 of ∼40 μM, and essentially no synergic activity with CBD on acute lymphocytic leukemia.^25^

However, synergism between plant-produced compounds was previously suggested because, in some cases, unrefined content of the flower extract, with its different extracted compounds, may have an advantage over the activity of an isolated compound.^12,13^ Activity of the purified compounds comprising most of the fractions, that is, THCA in F7 and CBGA in F3, was synergistic as well. Nevertheless, *in vivo* experiment for validation of synergism would be necessary. Often only additive effects are observed *in vivo*. For example, cannabinoid CB1 antagonist and systemic cholecystokinin-1-induced (CCK1) receptor agonist had additive effects on CCK1-induced feeding suppression in rats, providing a framework for combined therapies.^26^ Should this be the case for the interactions between the identified fractions and compounds in the present study, that is, additive rather than synergistic interaction *in vivo*, combined treatment could be a potential improvement over standard of care alone, particularly because of the low toxicity profile of cannabinoids.

The cytotoxic activity of F7 or F7+F3-involved cell apoptosis supports other studies, which suggest that cannabis-derived compounds induce apoptosis.^27,28^ The evidence that F7 treatment led to S phase cell cycle arrest, whereas F7+F3 treatment led to G0/G1 arrest suggests that the synergistic interaction between F7+F3 leads to a different form of regulation on cell cycle progression, compared with only F7.

In the F7+F3 treatment, but not in the F7 treatment, the gene expression involved in G1/S transition was suppressed. The suppressed genes include *cyclin E2* (geneID: 9134), by ∼10-fold. *Cyclin E2* mRNA levels oscillate throughout the cell cycle and reach highest levels at the G1/S boundary; *cyclin E2* is considered rate limiting for G1 progression.^29^ Repression of *cyclin E2* in the F7+F3 treatment may account for the G1 arrest. Interestingly, expression of *cyclin E1* (geneID: 898) is also repressed by F7+F3 treatment (by a multiple of ∼2.5). Since *cyclin E1* and *cyclin E2* have some redundancy, suppression of both further explains the F7+F3 effect on G1 arrest. Moreover, overexpression of both *cyclin E2* and *cyclin E1* positively affects cell proliferation in some cancer cell lines.^30^ Their reduction might again suggest that the F7+F3 treatment has anticancer properties.

The Wnt family of secreted glycoproteins induces signaling involved in processes of cell proliferation, differentiation, and oncogenesis, including colon cancer and melanoma; more than 90% of CRCs involve β-catenin-dependent WNT signal transduction.^31^ F7+F3 treatment reduced most differentially expressed genes related to Wnt signaling pathways, including, by a factor of eight, *Wnt16* (geneID: 51384) that was previously shown to be involved in leukemia.^32^

Frizzled proteins are one of the major Wnt receptors^31^ and their blockage is one target for anticancer drugs that interfere with canonical Wnt signaling.^33^ The reduction of the expression of *Frizzled class receptor 1* (geneID: 8321) by F7+F3 treatment may again implicate this treatment with the downregulation of the Wnt pathway. *TCF7* expression is also significantly suppressed by the F7+F3 treatment. Wnt pathway activation leads to β-catenin accumulation and translocation to the nucleus, where under the control of T cell factor (TCF), it activates transcription of target genes.^31,34^

TNF-α was suggested to be a prominent effector of colon cancer development. For example, it has been shown that TNF-α treatment in cultured cells resulted in increased chromosomal instability, gene mutations, and amplification^35^ and that TNF-α is a prominent mediator of the initiation and progression of colitis-associated colon carcinogenesis.^36^

Also, elevation in colonic TNF-α leads to protransformational alterations of key components of the Wnt signaling pathway.^37^ Interestingly, in our study where cells are also treated with TNF-α, treatment with F7+F3 resulted in differential expression of genes related to the Wnt signaling, suggesting that this treatment may counteract TNF-α-induced CRC. Further studies are needed to examine this suggestion.

F7+F3 treatment induces several apoptosis-promoting genes, including the tumor suppressor p53 (geneID: 7157) transcription factor that causes cell cycle arrest and apoptosis. P53 signaling is often dysregulated in CRC; patients with the mutant p53 gene may be resistant to current therapies, leading to a poor prognosis.^38^ *TRAIL-R2* (TNFRSF10B; geneID: 8795) expression is also induced by F7+F3 treatment. It is a receptor for TRAIL to induce apoptosis, but not necroptosis in CRC cells.^39^ However, FLIP (CFLAR; geneID: 8837), which inhibits TRAIL and caspase 8-dependent apoptosis^40^ is upregulated by F7+F3 treatment as well.

PUMA (geneID: 27113, BCL2-binding component 3) BH3-only Bcl-2 family proteins is a p53 downstream target and acts as a mediator for different tumor suppression drugs that treat CRC.^41^ PUMA expression is induced by F7+F3 cell treatment and may indicate the potential to increase PUMA-regulated treatment of CRC by treatment with the combination of F7+F3 and the relevant drugs. In this case and others, however, additional functional tests *in vitro* and *in vivo* are needed to fully confirm and characterize of the pathways activated by F7+F3 cell treatment.

The F7 and F7+F3 treatments showed only relatively low cytotoxic activity on a normal colon cell line but were active on adenomatous polyps. On the one hand, these extracts do not specifically target colon cancer cells that are a caveat for potential therapy. On the other, since nearly every carcinoma begins with an adenoma,^42^ F3, F7, and F7+F3 could be potential candidates for chemopreventive agents to either prevent or suppress progression of neoplastic polyps. The ability of F7+F3 treatment to induce cell cycle arrest and cancer cell apoptosis further suggests that F7+F3 treatment may have therapeutic anticancer value.

## Supplementary Material

Supplemental data

Supplemental data

Supplemental data

Supplemental data

Supplemental data

Supplemental data

Supplemental data

## Abbreviations Used

ACF: aberrant crypt foci
ANOVA: analysis of variance
CBD: cannabidiol
CBDA: cannabidiolic acid
CBG: cannabigerol
CBGA: cannabigerolic acid
CBN: cannabinol
CRC: colorectal cancer
CI: combination-index
FACS: fluorescence-activated cell sorting
GC/MS: gas chromatograph/mass spectrometry
GO: gene ontology
HPLC: high-performance liquid chromatography
NCBI: National Center for Biotechnology Information
NT: nontreated
PBS: phosphate-buffered saline
PI: propidium iodide
THC: tetrahydrocannabinol
THCA: tetrahydrocannabinolic acid
TNF: tumor necrosis factor
