# Supplementary material for: Identification of Synergistic Interaction Between Cannabis-Derived Compounds for Cytotoxic Activity in Colorectal Cancer Cell Lines and Colon Polyps That Induces Apoptosis-Related Cell Death and Distinct Gene Expression
Source: Cannabis Cannabinoid Res. 2018 Jun 1;3(1):120–35. doi: 10.1089/can.2018.0010 (PMC6038055; doi:10.1089/can.2018.0010)
Supplement: Supplemental data [file Supp_Fig1.pdf]

Supplementary Data

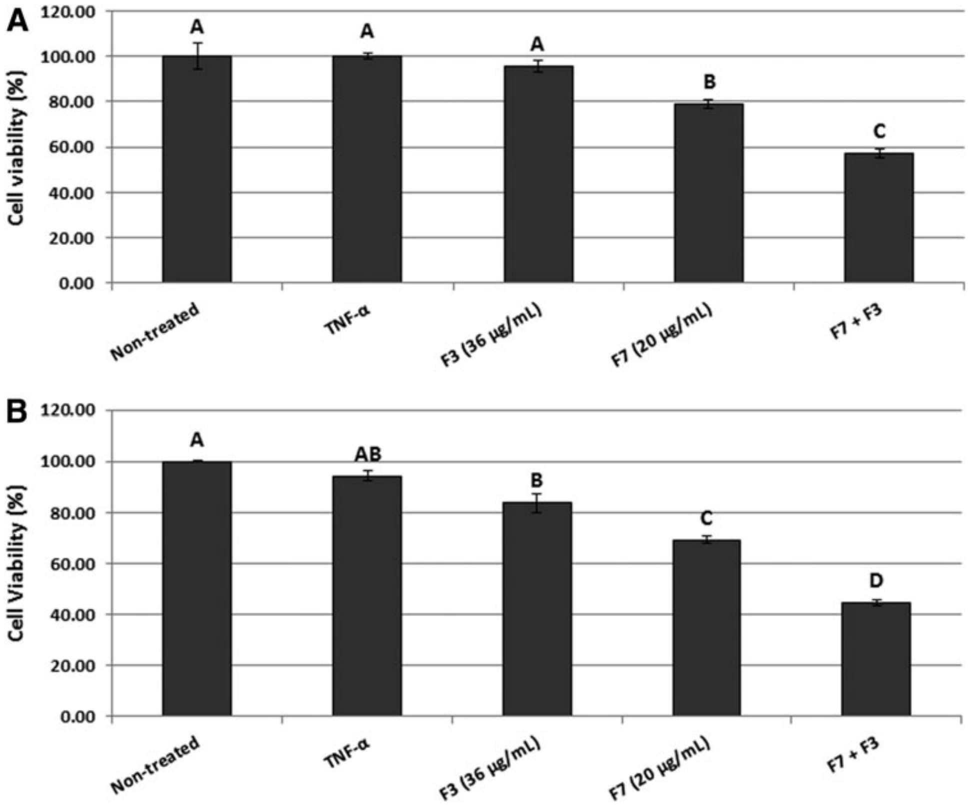

SUPPLEMENTARY FIG. S1. Effect of *Cannabis sativa* F7 and F3 on HT-29 **(A)** cell and Caco-2 **(B)** cell viability. Cell viability was determined using XTT assay as a function of live cell number. Cells were seeded and treated with IC50 doses of F3 (36 μg/mL), F7 (20 μg/mL), and the combination of F7 with F3 along with 50 ng/mL of TNF-α for 48 h. The cells were incubated next with XTT reagent for 2 h. Absorbance was recorded at 490 nm with 650 nm of reference wavelength. Values were calculated as the percentage of live cells relative to the nontreated control (cells without TNF-α and treatments) after reducing the absorbance without cells. Error bars indicate ±SE ($n = 3$). Levels with different letters are significantly different from all combinations of pairs by Tukey–Kramer HSD. HSD, honest significant difference; TNF, tumor necrosis factor.
