# Supplementary material for: Identification of Synergistic Interaction Between Cannabis-Derived Compounds for Cytotoxic Activity in Colorectal Cancer Cell Lines and Colon Polyps That Induces Apoptosis-Related Cell Death and Distinct Gene Expression
Source: Cannabis Cannabinoid Res. 2018 Jun 1;3(1):120–35. doi: 10.1089/can.2018.0010 (PMC6038055; doi:10.1089/can.2018.0010)
Supplement: Supplemental data [file Supp_Fig2.pdf]

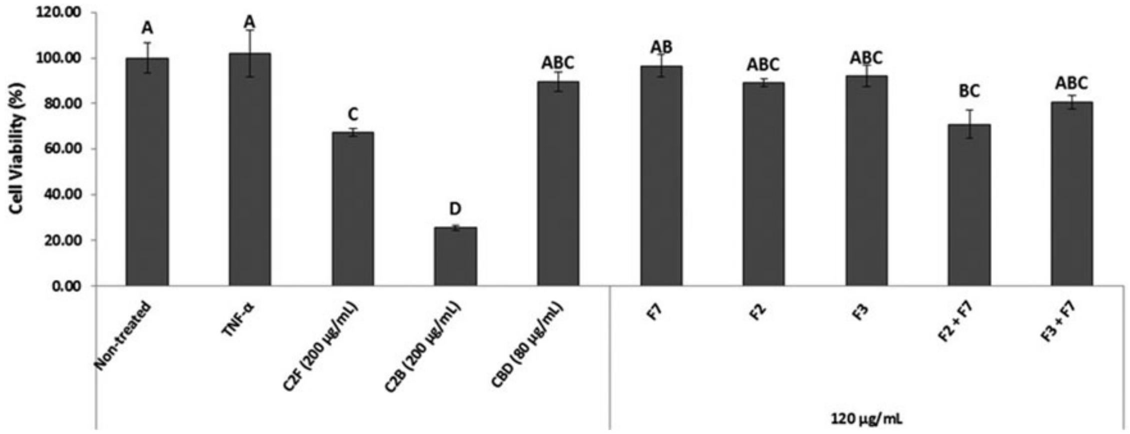


**SUPPLEMENTARY FIG. S2.** Effect of *Cannabis sativa* C2F, C2B and active fraction combinations on CCD-18Co healthy colon cell viability. Determination of CCD-18Co cell viability using alamarBlue fluorescence (Resazurin assay) as a function of live cell number. Cells were seeded and treated with *C. sativa* ethanol extracts of fresh inflorescences (C2F, 200 $\mu$g/mL), heated inflorescences (C2B, 200 $\mu$g/mL), F7 (120 $\mu$g/mL), F2 (120 $\mu$g/mL), F3 (120 $\mu$g/mL), and combinations of F7 with F2 and F3, along with 50 ng/mL of TNF-$\alpha$ for 16 h. The cells were then incubated with alamarBlue for 4 h. Relative fluorescence at the excitation/emission of 544/590 nm was measured. Values were calculated as the percentage of live cells relative to the nontreated (cells without TNF-$\alpha$ and treatments) control after reducing the autofluorescence of alamarBlue without cells. Error bars indicate ±SE ($n = 3$). Levels with different letters are significantly different from all combinations of pairs by Tukey–Kramer HSD.
