# Supplementary material for: Identification of Synergistic Interaction Between Cannabis-Derived Compounds for Cytotoxic Activity in Colorectal Cancer Cell Lines and Colon Polyps That Induces Apoptosis-Related Cell Death and Distinct Gene Expression
Source: Cannabis Cannabinoid Res. 2018 Jun 1;3(1):120–35. doi: 10.1089/can.2018.0010 (PMC6038055; doi:10.1089/can.2018.0010)
Supplement: Supplemental data [file Supp_Fig3.pdf]

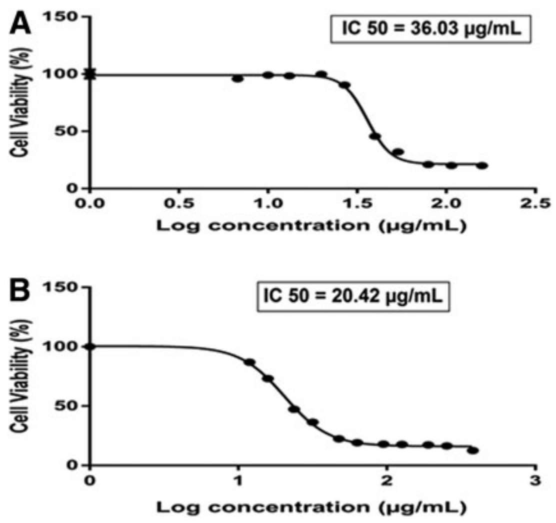

SUPPLEMENTARY FIG. S3. Determination of IC50 dose of F3 and F7. **(A)** Dose–effect curves of F3 (IC50 = 36.0 ± 0.6, 95% confidence interval = 35.0–37.1) on viability of HCT 116 colon cancer cells. Cells were seeded and treated with different concentrations of F3 (3–160 μg/mL) along with 50 ng/mL of TNF-α for 48 h. The cells were then incubated with XTT reagent for 2 h. Absorbance was recorded at 490 nm with 650 nm of reference wavelength. Values were calculated as the percentage of live cells relative to the nontreated control (cells without TNF-α and treatments) after reducing the absorbance without cells. For dose–response assays, data points were connected by nonlinear regression lines of the sigmoidal dose–response relation. GraphPad Prism was employed to produce dose–response curves and IC50 doses. **(B)** Dose–effect curves of F7 (IC50 = 20.4 ± 0.5, 95% Confidence interval = 19.7–21.1) on the viability of HCT 116 colon cancer cells. Cells were seeded and treated with different concentration of F7 (7.9–380 μg/mL) along with 50 ng/mL of TNF-α for 48 h. The cells were then incubated with XTT reagent for 2 h. Absorbance was recorded at 490 nm with 650 nm of reference wavelength. Values were calculated as the percentage of live cells relative to the nontreated (cells without TNF-α and treatments) control after reducing the absorbance without cells. For dose–response assays, data points were connected by nonlinear regression lines of the sigmoidal dose–response relation. GraphPad Prism was employed to produce dose–response curve and IC50 doses.
