# Supplementary material for: Identification of Synergistic Interaction Between Cannabis-Derived Compounds for Cytotoxic Activity in Colorectal Cancer Cell Lines and Colon Polyps That Induces Apoptosis-Related Cell Death and Distinct Gene Expression
Source: Cannabis Cannabinoid Res. 2018 Jun 1;3(1):120–35. doi: 10.1089/can.2018.0010 (PMC6038055; doi:10.1089/can.2018.0010)
Supplement: Supplemental data [file Supp_Table1.pdf]

Supplementary Table S1. Combination Index Analysis

A. F7 + F3			
CI	Fraction affected	Dose F3 (μg/mL)	Dose F7 (μg/mL)
0.87	0.79	36.00	63.00
0.75	0.79	36.00	47.50
0.63	0.78	36.00	31.70
0.78	0.67	36.00	23.80
0.71	0.66	36.00	15.80
0.73	0.61	36.00	11.90
0.72	0.58	36.00	7.90

B. F3 + F7			
CI	Fraction affected	Dose F3 (μg/mL)	Dose F7 (μg/mL)
0.81	0.83	80.00	20.00
0.66	0.80	53.30	20.00
0.78	0.67	40.00	20.00
0.72	0.61	26.67	20.00
0.69	0.56	20.00	20.00
0.72	0.48	13.30	20.00
0.80	0.40	10.00	20.00
0.95	0.30	3.67	20.00

CI calculated using CompuSyn software. Fraction affected (Fa) versus combination index were generated to determine the extent of synergy. (A) Constant F3 (36 μg/mL) with different concentrations of F7; (B) Constant F7 (20 μg/mL) with different concentrations of F3. Synergistic effects are defined as CI <1, additive effects are CI $= 1$, and antagonistic effects are CI >1.
