# Supplementary material for: Identification of Synergistic Interaction Between Cannabis-Derived Compounds for Cytotoxic Activity in Colorectal Cancer Cell Lines and Colon Polyps That Induces Apoptosis-Related Cell Death and Distinct Gene Expression
Source: Cannabis Cannabinoid Res. 2018 Jun 1;3(1):120–35. doi: 10.1089/can.2018.0010 (PMC6038055; doi:10.1089/can.2018.0010)
Supplement: Supplemental data [file Supp_Table2.pdf]

Supplementary Table S2. Combination Index Analysis

A. THCA + CBGA			
**Combination Index**	**Fraction affected**	**Dose CBGA (μg/mL)**	**Dose THCA (μg/mL)**
1.28	0.83	28	25
1.14	0.76	28	15
1.13	0.71	28	12
0.92	0.70	28	6
0.95	0.57	28	4

B. CBGA + THCA			
**Combination Index**	**Fraction affected**	**Dose CBGA (μg/mL)**	**Dose THCA (μg/mL)**
1.19	0.84	40	13.14
0.97	0.83	28	13.14
0.79	0.83	20	13.14
0.67	0.81	13.3	13.14
0.52	0.80	6.67	13.14

Fraction affected (Fa) versus combination index were generated to determine the extent of synergy. (A) Constant CBGA (28 μg/mL) with different concentrations of THCA; (B) Constant THCA (13.14 μg/mL) with different concentrations of CBGA. Synergistic effects are defined as CI <1, additive effects are CI = 1, and antagonistic effects are CI >1.

CBGA, Cannabigerolic acid; CI, combination index; THCA, Tetrahydrocannabinolic acid.
