# Supplementary material for: Identification of Synergistic Interaction Between Cannabis-Derived Compounds for Cytotoxic Activity in Colorectal Cancer Cell Lines and Colon Polyps That Induces Apoptosis-Related Cell Death and Distinct Gene Expression
Source: Cannabis Cannabinoid Res. 2018 Jun 1;3(1):120–35. doi: 10.1089/can.2018.0010 (PMC6038055; doi:10.1089/can.2018.0010)
Supplement: Supplemental data [file Supp_Fig4.pdf]

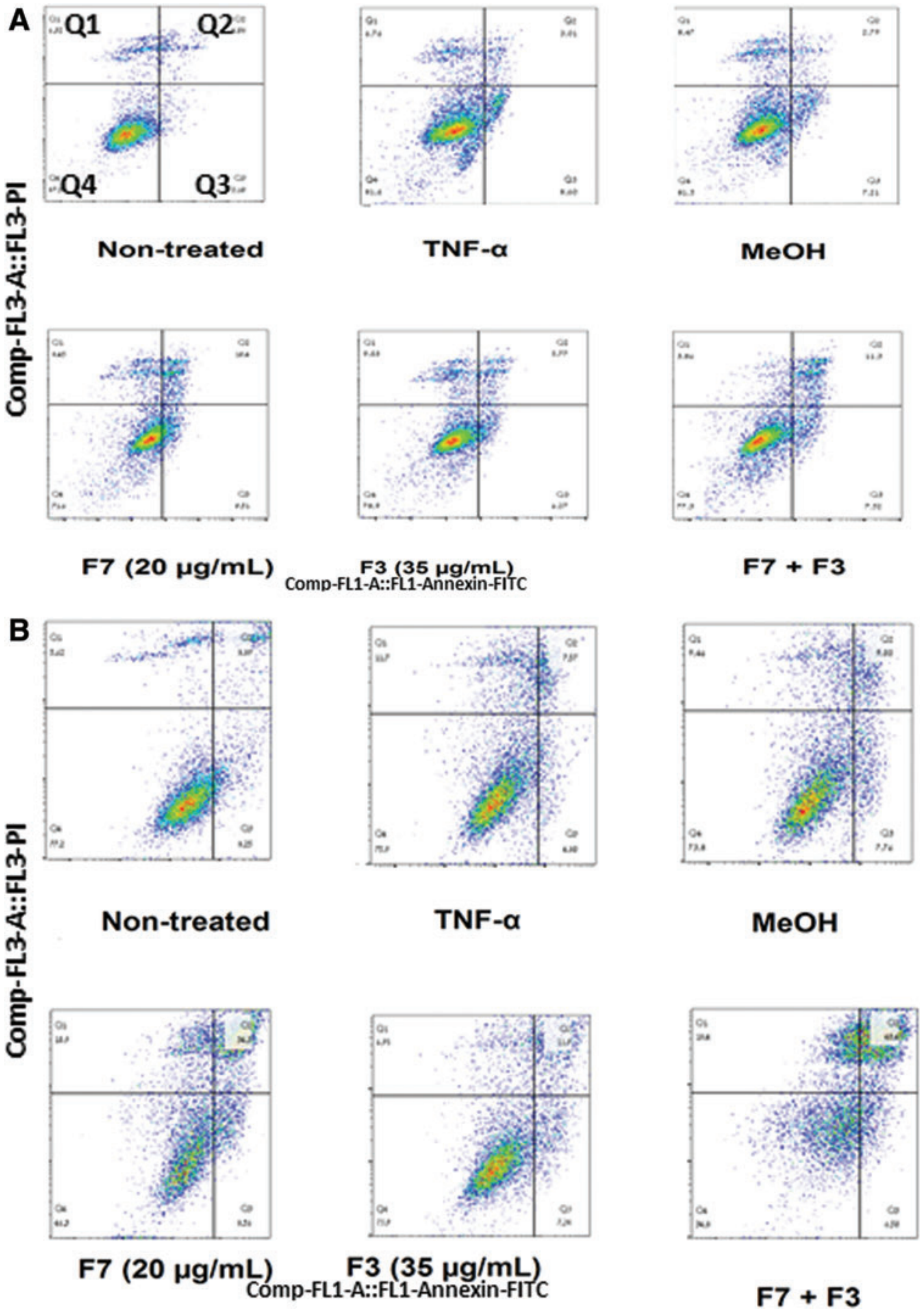

SUPPLEMENTARY FIG. S4. Determination of apoptosis or necrosis cytotoxic effect of F7, F3, and the combination of F7 with F3 on HCT 116 cells: output of FACS following Annexin V-FITC and PI staining. HCT 116 cells were treated with F7 (20 μg/mL), F3 (36 μg/mL), the combination of F7 with F3, and solvent control (methanol) along with TNF-α (50 ng/mL) for 24 h **(A)** or 48 h **(B)**. The treated cells were harvested and analyzed in FACS following Annexin V-FITC and PI staining. The histogram for each sample was split into four quadrants to indicate viable cells (lower left quadrant, Q4), early apoptotic cells (lower right quadrant, Q3), necrotic cells (upper left quadrant, Q1), and late apoptotic cells (upper right quadrant, Q2).
